# Impact of parity differences on residual feed intake estimation in Holstein cows

**DOI:** 10.3168/jdsc.2022-0307

**Published:** 2023-02-02

**Authors:** Ligia Cavani, Kristen L. Parker Gaddis, Ransom L. Baldwin, José E.P. Santos, James E. Koltes, Robert J. Tempelman, Michael J. VandeHaar, Malia J.M. Caputo, Heather M. White, Francisco Peñagaricano, Kent A. Weigel

**Affiliations:** 1 Department of Animal and Dairy Sciences, University of Wisconsin, Madison 53706; 2 Council on Dairy Cattle Breeding, Bowie, MD 48824; 3 Animal Genomics and Improvement Laboratory, Agricultural Research Service, USDA, Beltsville, MD 20705; 4 Department of Animal Sciences, University of Florida, Gainesville 32608; 5 Department of Animal Science, Iowa State University, Ames 50011; 6 Department of Animal Science, Michigan State University, East Lansing 48824

## Abstract

Residual feed intake (RFI) has been used as a measure of feed efficiency in farm animals. In lactating dairy cattle, RFI is typically obtained as the difference between dry matter intake observations and predictions from regression on known energy sinks, and effects of parity, days in milk, and cohort. The impact of parity (lactation number) on the estimation of RFI is not well understood, so the objectives of this study were to (1) evaluate alternative RFI models in which the energy sinks (metabolic body weight, body weight change, and secreted milk energy) were nested or not nested within parity, and (2) estimate variance components and genetic correlations for RFI across parities. Data consisted of 72,474 weekly RFI records of 5,813 lactating Holstein cows collected from 2007 to 2022 in 5 research stations across the United States. Estimates of heritability, repeatability, and genetic correlations between weekly RFI for parities 1, 2, and 3 were obtained using bivariate repeatability animal models. The nested RFI model showed better goodness of fit than the nonnested model, and some partial regression coefficients of dry matter intake on energy sinks were heterogeneous between parities. However, the Spearman's rank correlation between RFI values calculated from nested and nonnested models was equal to 0.99. Similarly, Spearman's rank correlation between the RFI breeding values from these 2 models was equal to 0.98. Heritability estimates for RFI were equal to 0.16 for parity 1, 0.19 for parity 2, and 0.22 for parity 3. Repeatability estimates for RFI across weeks within parities were high, ranging from 0.51 to 0.57. Spearman's rank correlations of sires' breeding values were 0.99 between parities 1 and 2, 0.91 between parities 1 and 3, and 0.92 between parities 2 and 3. We conclude that nesting energy sinks within parity when computing RFI improves model goodness of fit, but the impact on the estimated breading values appears to be minimal.

## Highlights

• Nesting energy sinks within parity when computing RFI provides superior fit.
• Minimal re-ranking of RFI phenotypes using nested or not nested (constant) models.
• Strong genetic correlation estimates among RFI across parities 1, 2, and 3.
• Minimal re-ranking of sires' breeding values for RFI in parities 1, 2, and 3.

## Graphical Abstract

**Figure:**
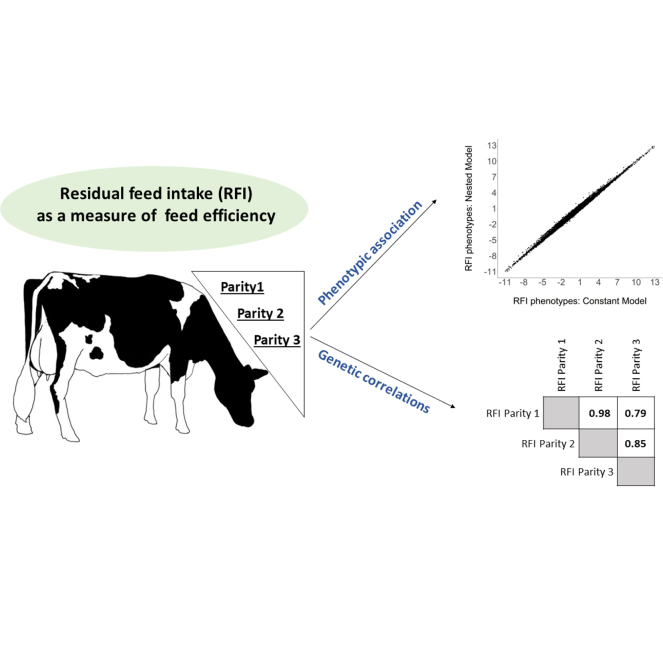
**Summary:** Residual feed intake (RFI) is typically obtained as the difference between dry matter intake (DMI) observations and predictions from regression on known energy sinks, and effects of parity, days in milk, and cohort. Understanding the impact of parity on RFI estimation is important because variation across parities might affect the estimation of RFI phenotypes and, hence, the prediction of RFI breeding values. To investigate this, we calculated RFI considering the energy sinks nested or not nested (constant) within parity using weekly records of 5,813 lactating Holstein cows collected from 2007 to 2022 in the United States. In addition, genetic correlations between weekly RFI for parities 1, 2, and 3 were estimated using bivariate repeatability animal models as the genetic breeding values for RFI in each parity. Our results showed that nesting energy sinks within parity when computing RFI improves model goodness of fit, but the impact on the RFI phenotypes and the estimated breeding values appears to be minimal.

Identifying cows that can convert feed to milk more efficiently than their contemporaries is challenging due to the biological complexity of feed efficiency. Residual feed intake (**RFI**) has been used widely as a measure of feed efficiency in cattle, and in lactating dairy cows it is usually obtained from regression of observed DMI on known energy sinks, namely metabolic body weight (**mBW**), body weight change (**ΔBW**), and secreted milk energy (**MilkE**). In addition, most RFI models account for parity, DIM, and cohort effects. The residual portion of this model represents the RFI phenotypes (Berry and Crowley, 2013; Tempelman et al., 2015; VandeHaar et al., 2016). Therefore, for a given population, more efficient cows eat less than predicted and have lower RFI values compared with the average of their contemporaries.

In general, RFI estimation models consider parity effects independent of the energy sinks (i.e., partial regression coefficients of DMI on energy sinks are constant across parities). However, these partial regression coefficients could vary across parities, particularly for primiparous cows that are still growing, and ignoring these differences might affect the estimation of RFI phenotypes, and hence, the prediction of RFI breeding values. The objectives of this study were to evaluate the impact of parity on RFI estimation in lactating Holstein cows by comparing RFI models considering energy sinks nested within parity or constant across parities, and to estimate genetic variance components and genetic correlations for RFI across different parities.

Data consisted of 72,474 weekly RFI records of 5,813 lactating Holstein cows (37,211 records of 3,584 cows in parity 1, 23,836 records of 2,324 cows in parity 2, and 11,427 records of 1,110 cows in parity 3) collected from 2007 to 2022 at 5 research stations: Iowa State University (Ames, IA), Michigan State University (East Lansing, MI), University of Florida (Gainesville, FL), University of Wisconsin–Madison (Arlington, WI), and USDA-Agricultural Research Service Animal Genomics and Improvement Laboratory (Beltsville, MD). All procedures were approved by the corresponding Animal Care and Use Committees. Animals were housed in freestall or tiestall facilities, and daily feed intakes were measured via roughage intake control system (Hokofarm Group), Calan Broadbent Feeding System (American Calan), GrowSafe System (Vytelle), or manual weigh-back of refusals. Milk weights were obtained daily, milk samples were obtained weekly for determination of milk composition, and BW were obtained on 3 consecutive days at the beginning, middle, and end of the experimental period. Milk energy (MilkE; Mcal) was calculated weekly using the following equation (NRC, 2001):

MilkE = (0.0929 × fat % + 0.0563 × protein % + 0.0395 × lactose %) × milk yield.

Since the experiments lasted only a few weeks, weekly BW were estimated using a simple linear regression of measured BW on days for each experiment. The mBW was calculated as weekly average BW^0.75^, and ΔBW was calculated as the difference in predicted BW at the end and beginning of each week.

Weekly RFI phenotypes were calculated using 2 alternative linear mixed models:

DMI = DIM + Lact + *b_1_*MilkE + *b_2_*mBW + *b_3_*ΔBW + block + week + e, [constant model]

DMI = DIM (Lact) + *b_1_*MilkE (Lact) + *b_2_*mBW (Lact) + *b_3_*ΔBW (Lact) + block + week + e, [nested model]

where DIM represents the effect of days in milk with 9 levels (grouped by 16 d), Lact represents the effect of parity (lactation number) with 3 levels (1, 2, and 3), MilkE is secreted milk energy with partial regression coefficient *b_1_*, mBW is metabolic body weight with partial regression coefficient *b_2_*, ΔBW is the change in body weight with partial regression coefficient *b_3_*, block represents the random effect of experiment-treatment, week represents the random effect of week of experiment, and e is the random residual of the model, representing RFI. Random effects were assumed to follow a multivariate normal distribution, with block $N\left(0,I\sigma_{block}^{2}\right),$ week $N\left(0,I\sigma_{week}^{2}\right),$ e $N\left(0,I\sigma_{e}^{2}\right),$ where **I** is the identity matrix, and covariances between these effects equal to zero. Therefore, the difference between these 2 alternative models consisted of nesting regression coefficients for DIM and the 3 energy sinks within parity in the nested model, versus treating them as constant across parities in the constant model. The mean and standard deviation of RFI were 0 ± 2.0 kg in both models, whereas the means and standard deviations of DMI, DIM, MilkE, mBW, and ΔBW were 24.8 ± 4.2 kg, 115.0 ± 37.6 d, 29.9 ± 5.8 Mcal, 126.0 ± 11.8 kg^0.75^, and 2.7 ± 3.0 kg, respectively. Goodness of fit was assessed using Bayesian information criterion and Akaike information criterion. We also evaluated the Spearman's rank correlation coefficient between RFI phenotypes from the 2 models, and compared partial regression coefficients for energy sinks across parities.

The impact of parity on RFI estimation was also evaluated in a genetic context. Weekly RFI phenotypes were calculated fitting a separate RFI model within each parity (1, 2, and 3) using the constant model described above, but with no parity effect as RFI within each parity was considered a unique trait. Estimates of heritability, repeatability, and genetic correlations between weekly RFI values for parities 1, 2, and 3 were performed using a bivariate repeatability animal model:

**y** = **Xβ** + **Zu** + **Wpe** + **e**,

where **y** is a vector of weekly RFI records, **β** is a vector of fixed effects of week, **u** is a vector of random additive genetic effects, **pe** is a vector of random permanent environmental effects, and **e** is the vector of random residual effects. Matrices **X**, **Z**, and **W** are incidence matrices relating **y** to **β**, **u**, and **pe**, respectively. Random effects were assumed to follow a multivariate normal distribution,

$$\left[\begin{array}{c} u\\ pe\\ e \end{array}\right]\;N\left\{\left[\begin{array}{c} 0\\ 0\\ 0 \end{array}\right],\;\left[\begin{array}{ccc} G_{0}⊗\;A & 0 & 0\\ 0 & P_{0}⊗\;I & 0\\ 0 & 0 & R_{0}⊗\;I \end{array}\right]\right\},$$

where **G_0_** and **P_0_** are the additive genetic and permanent environmental (co)variance matrices, respectively; **A** is the matrix of additive relationships between animals based on 5 generations of pedigree information; **R_0_** is the residual (co)variance matrix, and **I** is an identity matrix with suitable dimensions. Variance component estimates were obtained using REML with the AIREMLF90 software (Aguilar et al., 2018).

The Akaike information criterion and Bayesian information criterion values were 315,725 and 315,881, respectively, for the constant model, and 315,365 and 315,705, respectively, for the nested model, differing statistically by a chi-squared test (*P*-value = 2.2^−16^). Thus, the nested model provided superior fit. However, Spearman's rank correlation coefficient of RFI phenotypes obtained from the constant model and nested model indicated minimal re-ranking (Figure 1A, correlation = 0.99). In addition, Spearman's rank correlation between the RFI breeding values derived from these 2 models was equal to 0.98, showing also a minimal re-ranking for the estimated breeding values.

**Figure 1:**
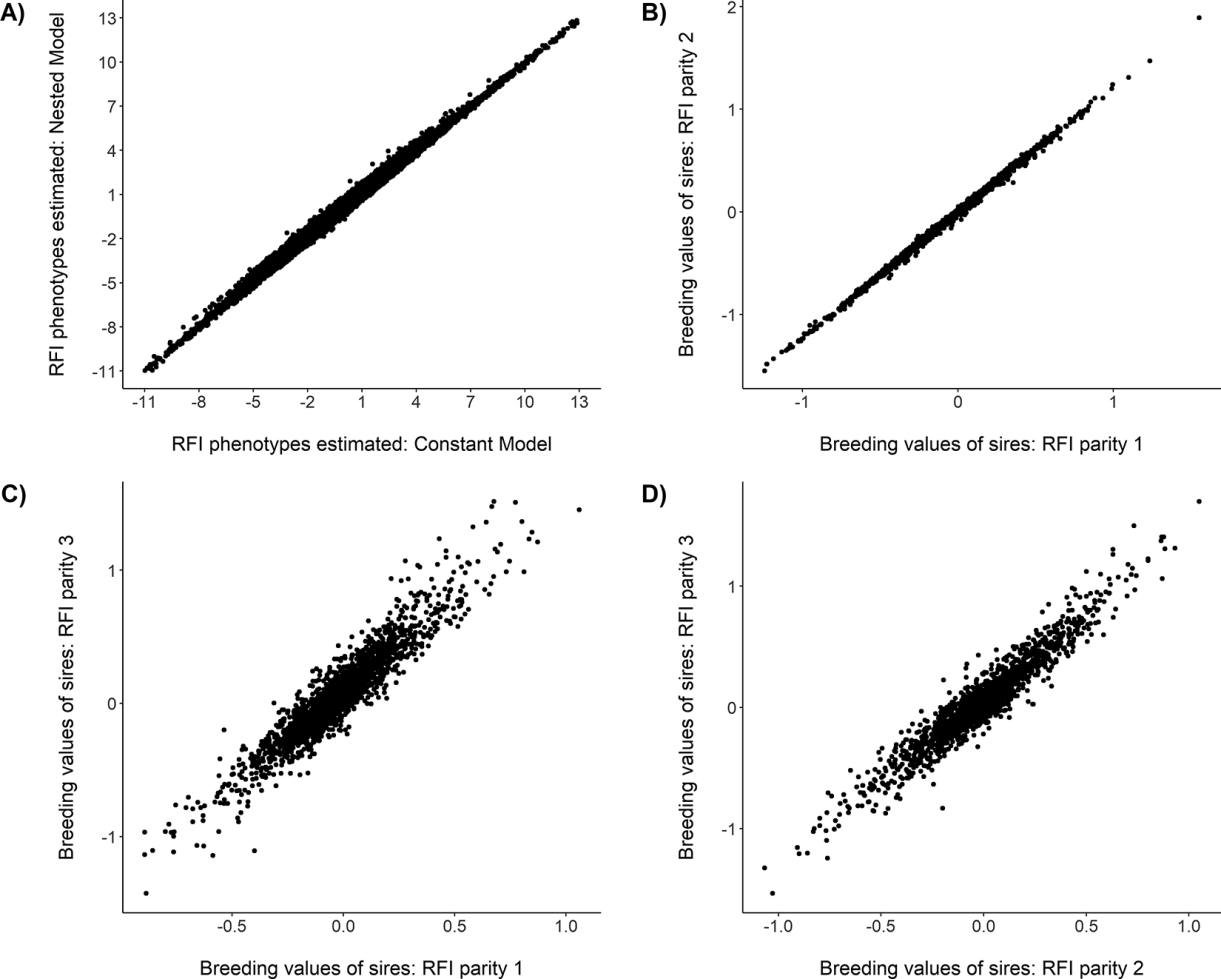
Relationship between residual feed intake (RFI) phenotypes calculated using the constant model and nested model (A); and between sires' RFI breeding values in parities 1 and 2 (B), parities 1 and 3 (C), and parities 2 and 3 (D).

Estimated partial regression coefficients of DMI on the energy sinks ranged from 0.376 to 0.382 kg of DMI per Mcal of MilkE, 0.107 to 0.112 kg of DMI per kg^0.75^ of mBW, and 0.086 to 0.124 kg of DMI per kg of ∆BW (Table 1). Based on the corresponding confidence intervals, some partial regression coefficients were heterogeneous across parities (nested model, Table 1). For MilkE, there was no difference in the partial regression coefficients across parities. However, for mBW, the partial regression coefficient for parity 2 were larger than that of parities 1 and 3, which were similar. Last, for ∆BW, the partial regression coefficient for parity 3 was significantly larger than that of parities 1 and 2, which were numerically similar. On average, partial regression coefficients were similar to those obtained in other feed efficiency studies in Holstein cows, many of which used overlapping data sets (Tempelman et al., 2015; Khanal et al., 2022), and also in a study using data from European research stations (Guinguina et al., 2020). Partial regression coefficients for DMI on ∆BW can be different across studies and difficult to compare due to differences in energy content of feed intake across populations/studies. As reported by Tempelman et al. (2015), variation in ∆BW regression coefficients seems to be greater than that of the other energy sinks when compared across models, data sets, and research stations, perhaps due to imprecision in ΔBW phenotypes measured over relatively short time periods. Negative partial regression coefficient for ∆BW was reported by Davis et al. (2014) in a study with lactating Holstein-Friesian cows.

**Table 1:** Estimated partial regression coefficients and 95% CI of DMI on each of 3 energy sinks (MilkE = milk energy, MBW = metabolic BW, and ΔBW = change in BW) based on the constant model and nested model

Item	MilkE (95% CI)	mBW (95% CI)	ΔBW (95% CI)
Constant model	0.378	0.110	0.097
	(0.374; 0.381)	(0.108; 0.112)	(0.091; 0.103)
Nested model			
Parity 1	0.382	0.107	0.095
	(0.377; 0.387)	(0.105; 0.110)	(0.087; 0.104)
Parity 2	0.380	0.112	0.086
	(0.375; 0.386)	(0.110; 0.114)	(0.077; 0.095)
Parity 3	0.376	0.108	0.124
	(0.369; 0.382)	(0.105; 0.110)	(0.111; 0.136)

The next step of our study was to evaluate potential genetic differences in RFI among parities. For this, we estimated genetic parameters and genetic correlations among parities 1, 2, and 3. Table 2 shows heritability, repeatability, and genetic correlation estimates for RFI in each of these 3 parities. Heritability estimates for RFI were equal to 0.16 ± 0.02 in parity 1, 0.19 ± 0.03 in parity 2, and 0.22 ± 0.06 in parity 3. Moreover, RFI phenotypes were repeatable across weeks in all parities, with repeatability estimates ranging from 0.51 to 0.57. Estimated genetic correlation for RFI were 0.98 ± 0.06 between parities 1 and 2, 0.79 ± 0.21 between parities 1 and 3, and 0.85 ± 0.18 between parities 2 and 3, indicating poorer precision for pairwise combinations involving parity 3. Complementing these results, Figure 1 (B, C, D) depict the relationship between sires' estimated breeding values for each parity, where the Spearman's rank correlation coefficient was 0.99 between parities 1 and 2, 0.91 between parities 1 and 3, and 0.92 between parities 2 and 3.

**Table 2:** Variance component and genetic parameter estimates (SE) for weekly residual feed intake (RFI) for parities 1, 2, and 3 in Holstein cows^1^

Parameter	RFI parity 1	RFI parity 2	RFI parity 3
Additive genetic variance	0.519 (0.08)	0.763 (0.14)	1.099 (0.31)
Permanent environmental variance	1.140 (0.07)	1.547 (0.12)	1.734 (0.28)
Residual variance	1.607 (0.01)	1.774 (0.02)	2.222 (0.03)
Heritability	0.16 (0.02)	0.19 (0.03)	0.22 (0.06)
Repeatability	0.51 (0.01)	0.57 (0.01)	0.56 (0.01)
$r_{g_{1,j}}$	1	0.98 (0.06)	0.79 (0.21)
$r_{g_{2,j}}$		1	0.85 (0.18)
$r_{g_{3,j}}$			1

1 *r_g_* = genetic correlations between parities 1,2; 1,3; and 2,3.

Given fixed experiment station resources for measurement of DMI, MilkE, mBW, ΔBW, and RFI phenotypes for national genetic evaluations of feed efficiency in dairy cattle, the structure of the data is limited, in terms of the number of cows in multiple lactations. Measurement of individual feed intake records is expensive and time consuming, and it is more valuable to measure phenotypes on a greater number of unique cows than to measure repeated phenotypes on the same cows in different lactations. The numbers of cows with repeated phenotypes in parities 1 and 2, parities 1 and 3, and parities 2 and 3 were 713, 222, and 266, respectively. This data structure resulted in larger standard errors (0.18 and 0.21) for the estimated genetic correlations between parities 2 and 3 and parities 1 and 3, and it may have contributed to the uncertainty of the genetic correlation (0.79) between parities 1 and 3, which presented the largest SE.

Despite the importance of feed efficiency in dairy cattle, research on the potential differences in RFI phenotypes and predicted breeding values across parities have been limited. Khanal et al. (2022) reported a difference in heritability estimates of RFI for primiparous (0.25 ± 0.08) and multiparous (0.17 ± 0.05) Holstein cows, as well as differences in the partial regression coefficients. Li et al. (2017) observed variation in partial regression coefficients and genetic variance components between different periods within the first lactation. In agreement with Lu et al. (2017), they noticed greater variation in early and late lactation, with only small differences between 50 and 200 DIM, the minimum and maximum used in the present study. Also, Martin et al. (2021b) observed that the averaged RFI over the lactation was highly correlated with RFI measured in mid lactation. Although our data are mainly from mid-lactation cows, future studies implementing flexible models, as random regression models, may be interesting to investigate the variation across time within lactation. Moreover, heritability estimates for RFI in lactating Holstein cows have been reported from 0.15 to 0.32 (Gonzalez-Recio et al., 2014; Tempelman et al., 2015; Khanal et al., 2022).

Overall, our findings suggest that the nested model is more elegant and provides greater goodness of fit, and it has the added advantage of providing a more detailed biological interpretation of differences in partial regression coefficients of DMI on known energy sinks within each parity. For these reasons, the nested model should be preferred for analysis of the effects of dietary treatments and other experimental interventions on RFI in lactating dairy cows (Martin et al., 2021a). However, considering regressions on the energy sinks as constant or nested within parity had a minimal impact on RFI phenotypes and resulted in negligible changes in cows' rankings by predicted breeding values. Strong and positive genetic correlations were observed between parities 1 and 2, and sires' predicted breeding values were relatively consistent across lactations, but our ability to draw conclusive inferences about genetic correlations between parities 1 and 3, and to a lesser extent parities 2 and 3, was hampered by the data structure in which repeated phenotypes of the same cows across multiple lactations were limited by costs and facility constraints.
